# Distinct Mechanisms of Multiple Alpha‐Band Activities in Frontal Regions Following an 8‐Week Medium‐ (Yoga) and High‐Intensity (Pamela) Exercise Intervention

**DOI:** 10.1111/cns.70405

**Published:** 2025-05-05

**Authors:** Kaixuan Shi, Huipeng Lei, Lulu Chen, Xiaojing Wang, Meijia Li, Naem Haihambo, Zhizhen Zhang, Xuehong Qu, Xueyang Li, Jiazheng Peng, Talifu Zikereya, Chuanliang Han

**Affiliations:** ^1^ Department of Physical Education China University of Geosciences Beijing China; ^2^ Faculty of Psychology and Center for Neuroscience Vrije Universiteit Brussel Brussels Belgium; ^3^ Centre for Human Brain Health, School of Psychology University of Birmingham Birmingham UK; ^4^ Department of Psychology and Center for Neuroscience Vrije Universiteit Brussels Brussels Belgium; ^5^ Department of Social Neuroscience, Faculty of Medicine Ruhr‐University Bochum Bochum Germany; ^6^ Research Center One Health Ruhr of the University Alliance Ruhr Bochum Germany; ^7^ Department of Mathematics and Statistics University of Massachusetts at Amherst Amherst Massachusetts USA; ^8^ School of Biomedical Sciences and Gerald Choa Neuroscience Institute The Chinese University of Hong Kong Hong Kong SAR China

**Keywords:** alpha‐band activity, EEG, exercise intensity, Pamela, yoga

## Abstract

**Aim:**

Long‐term moderate‐ to high‐intensity exercise has been shown to significantly enhance overall health such as the improvement of physiological indicators and brain functions. One key aspect of brain activity is alpha‐band activity, which encompasses various sub‐oscillations within the alpha frequency band. However, the precise functions of these alpha sub‐oscillations following different exercise regimens remain unclear.

**Methods:**

We recruited 58 healthy college students and divided them into four groups: Pamela (high‐intensity interval training, HIIT), yoga (moderate‐intensity continuous training, MICT), and their corresponding matched control group (no exercise) for each exercise intervention group. Participants in the exercise intervention groups underwent training for up to 8 weeks (HIIT or MICT). Resting‐state EEG data were collected before and after training, both with eyes open and closed.

**Results:**

Following HIIT, the Pamela group experienced a significant reduction in body fat percentage and a notable increase in skeletal muscle mass. In terms of neural activity, the main difference was observed in the mid‐frequency alpha range in the frontoparietal region during the eyes‐open resting state. Conversely, after 8‐week yoga training, participants demonstrated a significant improvement in the duration of maintaining balance and sleep quality, and the main neural difference was reflected in the low‐ and high‐frequency alpha band activities in the bilateral frontotemporal regions during the eyes‐closed resting state.

**Conclusion:**

This study, for the first time, differentiates the effects of long‐term moderate‐ and high‐intensity exercise on neural oscillation during different resting states, which highlights that different sub‐frequency bands within the alpha frequency band would represent different exercise‐related functions.

## Graphical abstract

The main difference was observed in the mid‐frequency alpha range in the frontoparietal region during the eyes‐open resting state. Conversely, after 8‐week yoga training, the main neural difference was reflected in the low‐ and high‐frequency alpha band activities in the bilateral frontotemporal regions during the eyes‐closed resting state.

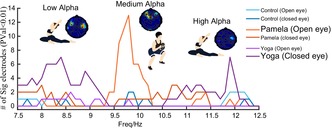

## 1 Introduction

Exercise is crucial for health, and regular physical activity can improve cognitive functions [1, 2], executive functions [3, 4, 5], and enhance the functions of multiple body systems, such as the musculoskeletal system [6], cardiovascular system [7, 8, 9], and respiratory system [10]. Different intensities of exercise exert different effects on the body. Many daily fitness activities among young people, such as yoga and Pamela, fall into the moderate‐ to high‐intensity category. Yoga, which is classified as a moderate‐intensity exercise (moderate‐intensity continuous training, MICT), emphasizes body flexibility, balance, and posture control [11] It contributes to improved body flexibility, strength, balance, core stability, and stress reduction [12]. Conversely, Pamela, which is high‐intensity interval training (HIIT) typically includes high‐intensity bursts of activity and short rest intervals [13]. HIIT can improve cardiorespiratory endurance, muscle strength, explosive power, and promote fat burning and weight management [11, 14, 15, 16]. Both long‐term moderate‐ and high‐intensity exercise yield distinct health benefits at different levels [17]. However, understanding how these health benefits are represented in the brain is not yet fully understood.

Research on changes in neural responses before and after exercise is crucial for understanding how exercise improves overall health [17]. Electroencephalography (EEG) is a common noninvasive method for recording neural activities [18, 19, 20, 21, 22, 23, 24]. Combining EEG with exercise‐related experimental paradigms can provide answers to such questions [25]. Neural oscillations are important components of neural electrical activity recorded by EEG [26, 27, 28, 29, 30], involving synchronous activities of neuron populations [31, 32, 33, 34, 35]. Among them, alpha oscillations (8–12 Hz) are a type of neural oscillation that reflects the internal state of the body and is highly related to human cognitive functions [36], such as attention [37, 38, 39, 40, 41, 42], memory [43, 44, 45, 46, 47, 48], and learning [49]. Studying alpha oscillations helps us understand the operational mechanisms in the brain before and after exercise, and how to use these principles to improve human health and cognitive capabilities. In the field of sport science, previous studies have found that individuals who engage in high‐intensity exercise tend to show increased alpha oscillation intensity and decreased peak frequency, etc. [38, 50, 51]. Similarly, in yoga exercise, previous research has found an increase in alpha wave production [52]. However, recent studies have unveiled the existence of multiple different alpha sub‐oscillations within the alpha frequency band [53]. These sub‐oscillations originate from different neural sources [30], yet their precise functions remain unclear. Currently, there is a gap in research regarding the effects of different types of exercise interventions on different alpha sub‐oscillations.

This study aimed to investigate the relationship between brain electrical alpha oscillations and long‐term moderate‐ to high‐intensity exercise. To achieve this, we designed two separate experimental comparisons: (1) a yoga group with its matched control group, and (2) a HIIT group with its matched control group. Each control group was matched for age and gender with its corresponding exercise group. After an 8‐week training program (yoga, HIIT, or control), we explored the significant alterations in brain electrical alpha oscillations at different levels of exercise intensity under different fine‐frequency conditions.

## 2 Materials and Methods

### 2.1 Participants

A total of 58 participants took part in the experiment. Sixteen participants (Age = 19.19 ± 1.97, 14 Male, two Female) were in the Pamela group (HIIT) and were matched with 13 participants in the corresponding control group in terms of age and gender (Age = 20.23 ± 2.12, 12 Male, one Female). Fifteen participants (Age = 19.2 ± 0.56, 15 Female) were in the MICT group (Yoga) and were matched with 14 participants in the control group based on age and gender (Age = 19.35 ± 1.39, 14 Female). The participants in the two experimental groups were recruited separately, and they were individuals who had not engaged in regular exercise in the past 3 months. Due to the higher intensity of HIIT exercises, there was more interest from male participants. On the other hand, yoga exercises are gentler, so all participants who joined the experiment were females. Then, in order to match the gender distribution in the two experimental groups, we maintained consistency when recruiting participants for the control group. This study adhered to the principles outlined in the Declaration of Helsinki and was approved by the Beijing Sport University Ethics Committee (Sports Science Experiment) (Approval number: 2020073A).

### 2.2 MICT Training Design

Based on previous research and the recommended physical activity guidelines from the World Health Organization [25], this study used traditional Hatha yoga as the intervention method, with 90‐min sessions three times (individual fixed time) a week for a total of 8 weeks. The 90‐min program consists of three components: 10 min of breathing training, 65 min of posture training, and 15 min of meditation. (1) Breathing training: focusing on abdominal breathing, supplemented by chest breathing and complete breathing. The ratio of inhalation to exhalation was no less than 3 s for both phases. The aim was to foster a natural and uniform breathing without holding the breath. During this phase, participants were encouraged to place their hands on either their abdomen or chest. Abdominal breathing aimed to enhance awareness of abdominal expansion and contraction with the breath. Chest breathing focused on the expansion and contraction of the chest cavity in all directions, and complete breathing was introduced in the last 2 weeks, after participants had mastered abdominal and chest breathing completely and independently. (2) Posture: The sequence of posture practice followed the order of supine‐prone‐kneeling‐squatting‐standing. This training focused on eight themes, including leg, abdominal, and back muscle strength improvement, hip, shoulder, and hamstring flexibility, balance establishment, and the sun salutation series, progressing in that order. This sequence aimed to improve neuromuscular activity and joint flexibility while building foundation strength, ultimately promoting balance improvement. (3) Meditation: The meditation session was conducted in a completely relaxed supine posture, guided by a professional yoga teacher with over 7 years of experience in voice meditation. The teacher was required to observe the participants' facial expressions and eye movements to ensure that meditation was conducted in a state of physical relaxation and focus.

Balance was tested using the “Eagle Pose”, which required participants to stand on one‐leg standing, with arms intertwined and eyes closed [54]. Here are the step‐by‐step instructions for conducting this test: (1) Determine the test site: Choose a spacious and flat ground, ensuring that there are no obstacles or debris that may cause falls. (2) Determine the test position: Stand on flat ground with both feet initially on the ground. Lift one foot, raise both arms laterally, and then bend forward by the waist. Extend the non‐supporting leg backward, maintaining a position in which the trunk and non‐supporting leg are parallel to the ground. (3) Eye closure: Close your eyes and maintain the closed‐eyed state. (4) Posture maintenance: Try to maintain a stable position, keeping your balance, and avoiding any contact with the ground or reliance on other support. (5) Timing: Use a timer or stopwatch to record the duration of the test, which should be a duration of maintaining a stable position per side. (6) Test the other foot: After completing the test on the first foot, repeat the same steps to test the balance on the other foot. (7) Record the results: Record the test duration for each foot, as well as any imbalances or swaying situations by the teacher. Calculate the average time as the final balance time measurement. Better balance ability is reflected in a longer duration of maintaining stability during the test.

The Pittsburgh Sleep Quality Index (PSQI) [55] was used to evaluate the sleep quality of participants. Select sleep quality, time to fall asleep, sleep duration, sleep disorders, daytime dysfunction, and total sleep score as evaluation indicators. Each factor is scored in four levels based on a score of 0–3. The cumulative score for each factor is the PSQI total score (0–21 points), with a higher total score indicating poorer sleep quality, 0–5 points indicating good sleep quality, 6–10 points indicating decent sleep quality, 11–15 points indicating average sleep quality, and 16–21 points indicating poor sleep quality.

### 2.3 HIIT Training Design

Five days before HIIT, all participants visited the sports physiology laboratory for maximal oxygen consumption (VO_2_max) testing to measure aerobic fitness and cardiovascular endurance and determine their maximum active heart rate. Before and after the intervention, various assessments were conducted, including body composition tests, 20‐m shuttle run tests (20 m SRT), and the collection of electroencephalograms (EEG) data. During the subsequent HIIT intervention, participants maintained their regular daily diet and rest, and their existing lifestyle was not disturbed except for the exercise program. The HIIT training was conducted by two professional fitness coaches with more than 4 years of teaching experience. To ensure safety, real‐time heart rate monitoring was employed during the entire HIIT process using Polar H10 heart rate belts. If the participant's heart rate exceeded 95% of their maximum heart rate, another coach guided the participant to reduce the exercise intensity. Meanwhile, the control group received weekly follow‐ups from another staff member for physical activity assessment.

To conduct the intervention, we adopted on‐site supervision training measures. Participants underwent HIIT training sessions within a specialized sports training room on the campus of China University of Geosciences. The training courses started at a fixed time for each participant each week, lasting approximately 30–35 min. The courses included warm‐up preparations (5 min), formal training (20 min), and relaxation exercises (over 5 min). The formal HIIT program consisted of 40‐s high‐intensity interval exercises at a speed of 85%–90% of their maximum heart rate, followed by 20 s of active recovery, maintaining a heart rate range of 60%–75% of the maximum heart rate. After the formal training, participants had relaxation exercises for over 5 min to cool down and recover. The HIIT program was conducted 3 times weekly for a total of 8 weeks. During the training, coaches provided exercise guidance and instructions to participants. In addition, participants were encouraged to exchange their experiences and achievements with each other to motivate one another. In the first 2 weeks, the focus of the HIIT intervention was primarily on bodyweight exercises, mainly to promote cardiovascular adaptation. The HIIT plan was designed based on the Pamela and Lemme exercises. These exercises were categorized as progressive training, including weight resistance exercises and equipment resistance exercises. The equipment used included dumbbells (5 kg), fitness balls (5 kg), and kettlebell exercises (5 kg). Body fat and muscle mass measurements were taken from all participants using the body composition analyzer (In‐body 570) both before and after the training period.

### 2.4 Electrophysiological Recordings

We used the Poseidon next‐generation brain neural activity monitoring system developed by Bio‐Signal Technologies for EEG data acquisition. The system has 32 recording channels for wireless data transmission. Before data acquisition, participants first washed and dried their hair to reduce impedance at the electrode‐scalp interface. Then, an electrode cap was worn with the nose root, occipital protuberance, and left and right preauricular points as references, ensuring that the Cz electrode is in the center. The Fz and other midline electrodes were aligned with the nasal root. The front edge of the electrode cap was positioned slightly above the eyebrows, and it was tightly secured on the scalp. After applying, we injected electrode paste at each electrode position to ensure an impedance of less than 5KΩ. Then, acquisition parameters were configured, with a sampling frequency set at 500 Hz. All channels were average referenced. The quality of the data is confirmed by checking the fluctuations in the data from the electrodes in the frontal lobe area during blinking and the occipital lobe area electrodes during eye closure. We evaluated the quality of the EEG signal by impedance method. Ocular artifacts were removed from the data using semiautomatic Independent Component Analysis using eeglab [56]. The extracted independent components were manually inspected, and ocular artifacts were removed by back‐projecting all components except those containing artifacts.

### 2.5 Data Filtering

All data were analyzed using custom codes written in MATLAB (The MathWorks, R2020a). The original continuous data were high‐pass filtered at 0.5 Hz and low‐pass filtered at 20 Hz, with zero‐phased FIR filters that filter the data both forward and backward to ensure phase delays introduced by each filter are nullified.

### 2.6 Power Spectrum Analysis

Data processing was performed in MATLAB (www.mathworks.com) with custom scripts. We used spectrum analysis to quantify the alpha oscillation strength in all electrodes. Similar methods have been applied in various biomedical fields, such as life sciences [26, 28, 34, 57, 58], neuropsychological disorders [27, 29, 30, 59], and other diseases [60, 61, 62, 63, 64, 65, 66], etc. The power spectral density (PSD) was computed using the multi‐taper method with 5 tapers using the Chronux toolbox [67], an open‐source data analysis toolbox (Chronux) available at http://chronux.org. Relative power was calculated by dividing the power at a specific frequency by the total power summation from 5 to 20 Hz.

### 2.7 Statistical Analysis

**FIGURE 1:**
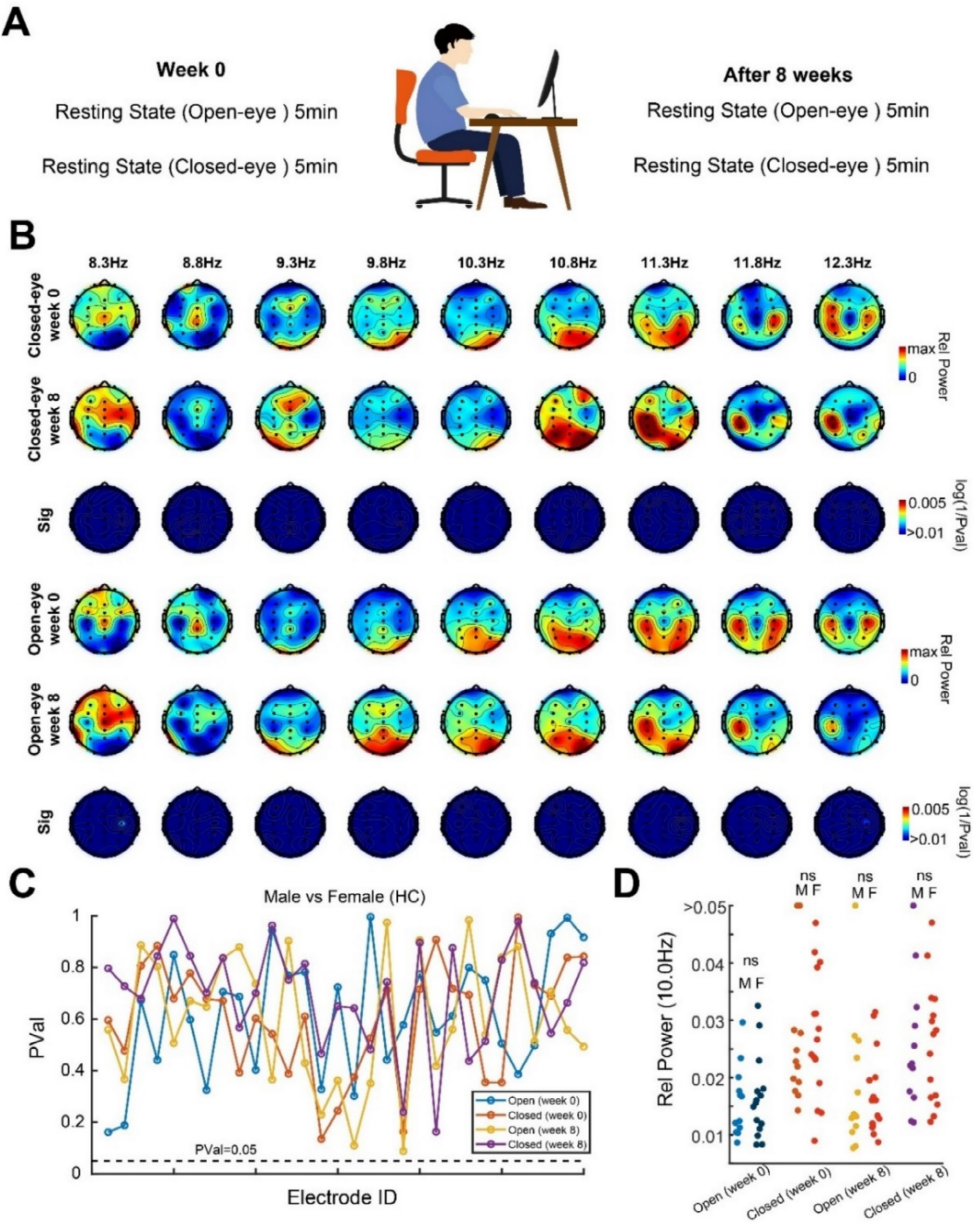
Comparison of alpha‐band activities in week 0 and week 8 in the control group. (A) Scheme of EEG recording for all subjects in control groups. (B) Topographic maps of both control groups in closed (rows 1 and 2) and open eyes (rows 4 and 5) states in the alpha frequency band in week 0 and 8, and their comparison significance (rows 3 and 6). (C) Significance comparison between males and females in the control group for alpha power in all recording electrodes. (D) Scatter plot for the comparison of relative power in closed and open eye state in the alpha frequency band in weeks 0 and 8.

**FIGURE 2:**
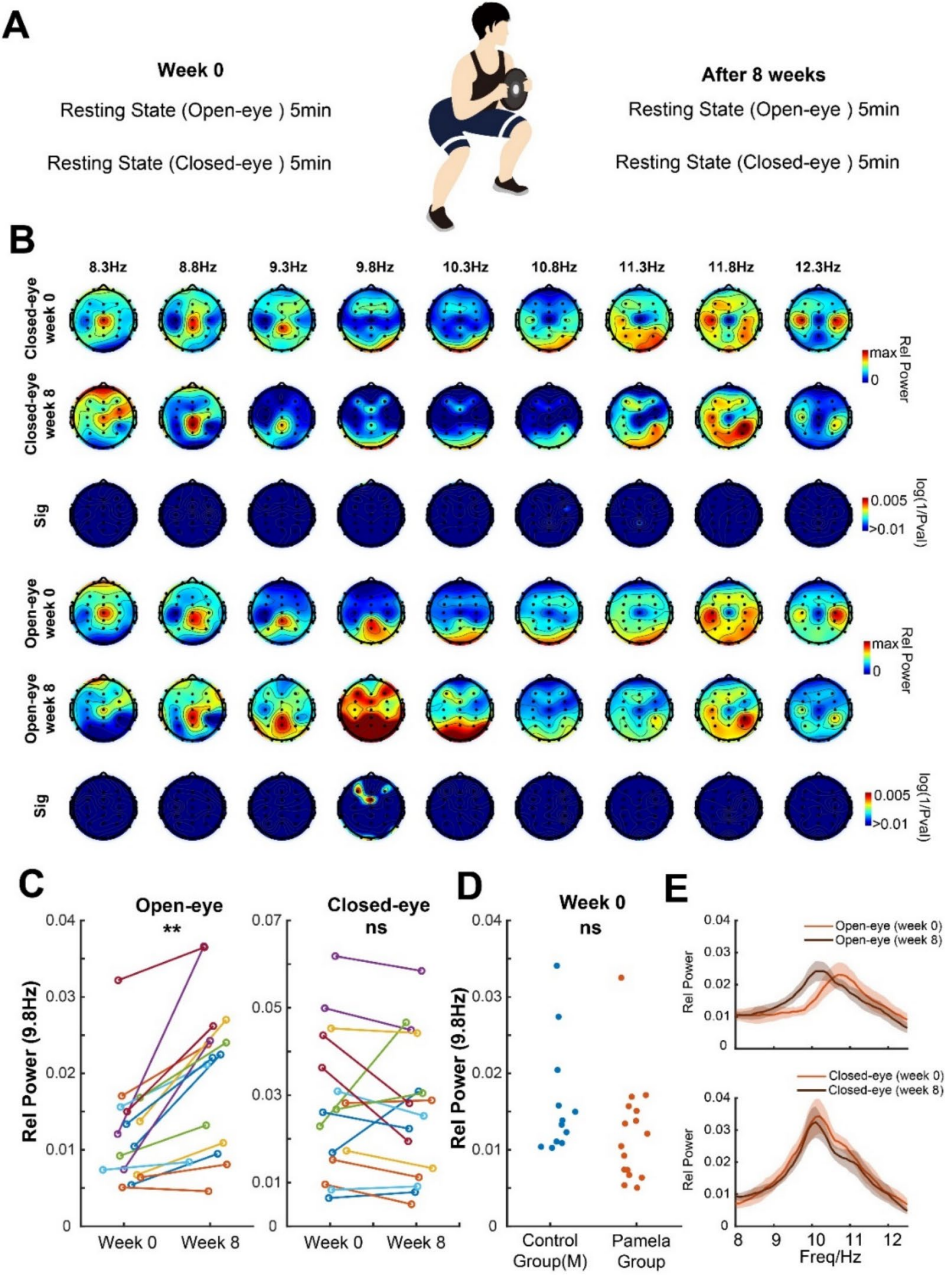
Comparison of alpha‐band activities in week 0 and 8 in HIIT group. (A) Scheme of EEG recording for the HIIT group before and after 8‐week training. (B) Topographic maps of the HIIT group in closed (rows 1 and 2) and open eye (rows 4 and 5) states in the alpha frequency band in week 0 and week 8, and its comparison significance (rows 3 and 6). (C) Change of relative medium alpha power in open and closed eye states. (D) Comparison between relative medium alpha power between HIIT and the corresponding control group in week 0. (E) Power spectrums in closed‐ and open eye states in the alpha frequency band in week 0 and week 8, shaded area is SEM.

**FIGURE 3:**
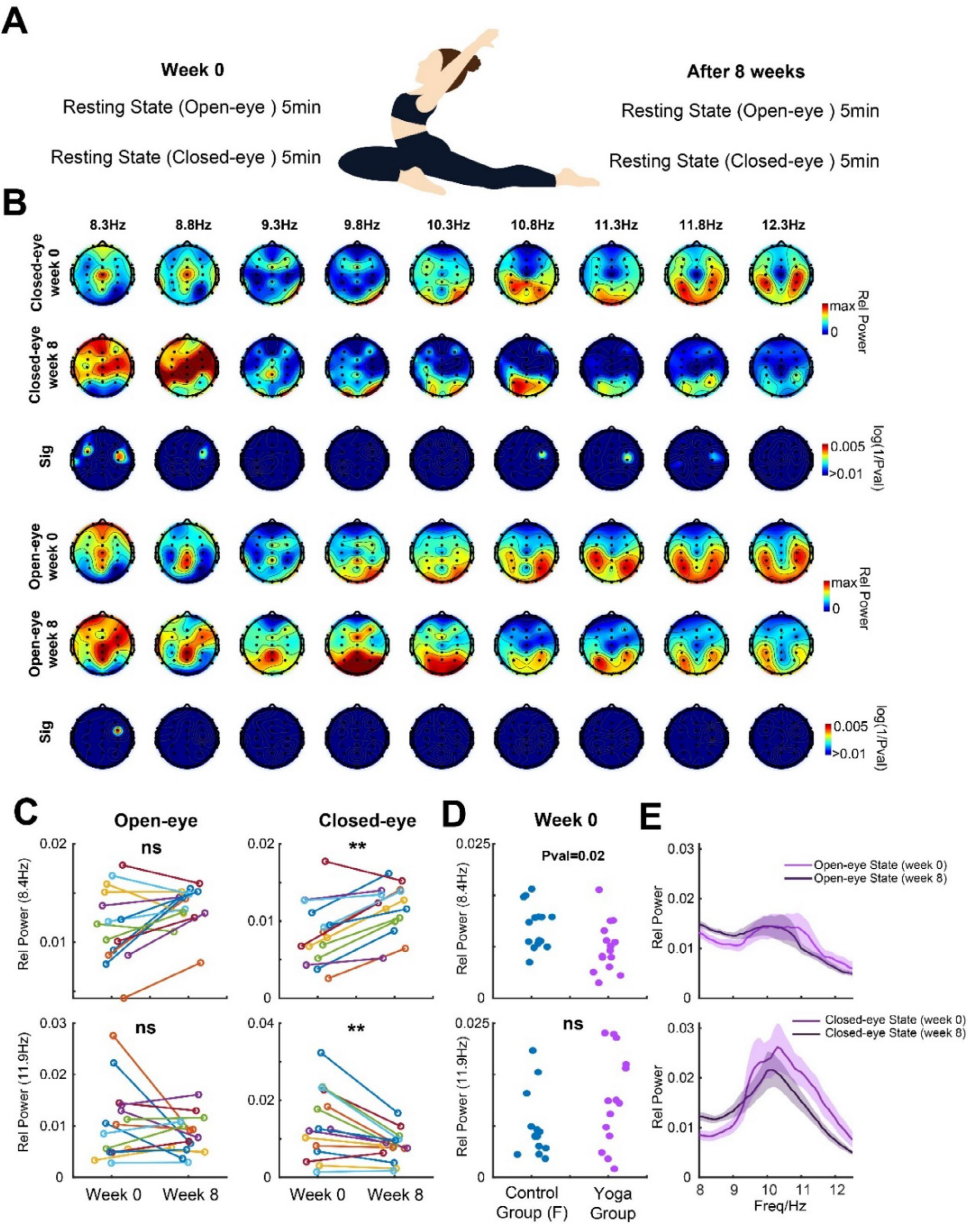
Comparison of alpha‐band activities in week 0 and week 8 in the MICT group. (A) Scheme of EEG recording for the MICT group before and after 8‐week training. (B) Topographic maps of the MICT group in closed (rows 1 and 2) and open eye (rows 4 and 5) state in the alpha frequency band in week 0 and week 8, and its comparison significance (rows 3 and 6). (C) Change in low (row 1) and high (row 2) alpha power in open and closed eye states. (D) Comparison between relative medium alpha power between MICT and the control group in week 0. (E) Power spectrums in closed and open eye state in the alpha frequency band in week 0 and week 8 in the yoga group, shaded area is SEM.

A normality test was performed on each dataset using the Shapiro–Wilk test or D' Agostino and Pearson test. A pairwise *t*‐test for each electrode was conducted to examine the differences in alpha band power at two time points: week 0 (baseline) and week 8 (post‐intervention) separately for each group. The paired *t*‐test would be considered as a process of identifying regions of interest (ROIs) in alpha bands. We then fixed three ROIs. In the MA band, ROI‐1 is the significant region in the HIIT group in the MA band in the open‐eye state. In the LA band, ROI‐2 is the significant region in the MICT group in the closed‐eye state. In the HA band, ROI‐3 is the significant region in the MICT group in the HA band in the closed‐eye state. Then, we performed paired *t*‐tests again in the experimental and control groups in different ROIs and frequency bands, respectively, to obtain new *p* values (Figures 1D, 2C, 3C). Additionally, to examine the differences in alpha power between the experimental groups (HIIT or MICT) and their corresponding control groups at baseline (week 0), a *t*‐test was conducted. This test was to ensure any observed differences in alpha power were not due to differences in participant characteristics but rather attributed to the effects of the intervention itself. In terms of physical changes, we used a paired *t*‐test to test the change in body fat percentage, muscle mass, body balance duration, and PSQI before and after exercise training in four groups. Then, we used a *t*‐test to compare the difference in the change of these indicators between the experimental group and the control group.

## 3 Results

In order to study the relationship between multiple alpha‐band activities and medium‐to‐high‐intensity exercise, we divided participants into four groups (HIIT group, and yoga group, and their respective matched control groups) and collected their EEG (10–20 system) data before and after 8 weeks of training in both eyes‐open and eyes‐closed resting states for 5 min. For each EEG channel, the recorded data was segmented into 10‐s intervals, and then the power spectrum was estimated by a multi‐taper method with a frequency resolution of 0.1 Hz. To ensure that there were no statistically significant differences in daily physical activity levels among the groups before the exercise intervention, we estimated the activity levels using the International Physical Activity Questionnaire (IPAQ) questionnaire. A 1‐way ANOVA analysis revealed no significant differences among the four groups (*p* > 0.05). Additionally, after conducting *t*‐tests with correction, we found no significant differences between the HIIT group and the control group (*p* > 0.05), as well as between the MICT group and the control group (*p* > 0.05).

### 3.1 No Significant Changes in EEG Alpha‐Band Activities in the Control Group

We first compared the changes in neural activity in the control group before and after 8 weeks, during which the participants did not engage in any specific exercise arrangements and had the freedom to keep their regular activities (Figure 1A). Through a significant comparison of the alpha frequency band activity in their brain waves before and after this 8‐week period, we found that there were no significant differences in alpha frequency band power, regardless of whether they had their eyes open or closed (*n* = 27) (Figure 1B). Given that our subsequent exercise experiment groups had gender differentiation (with the MICT group mainly composed of females, and the HIIT group predominantly composed of males), we also compared potential gender‐based differences in alpha oscillations before and after the 8‐week period in the control group. However, we did not identify any significant gender differences in the alpha oscillations across all electrodes (Figure 1C), and the specific scatter plots for each person are shown in Figure 1D. However, for the sake of experimental rigor and to have better control over gender distribution in the experimental groups, we proceeded to compare the male participants in the control group with all participants in the HIIT group in the following results and the female participants in the control group with the MICT group.

### 3.2 Significant Increase in Medium Alpha Power in Open‐Eye State in the HIIT Group

The HIIT group underwent an 8‐week daily fixed‐intensity HIIT training (Figure 2A). We found that in the open‐eye state, there was a significant increase in the power of the mid‐frequency alpha band (~9.8 Hz, medium alpha) in the frontal‐central region (ROI‐1). No significant differences were found in other frequency bands or the closed‐eye state (Figure 2B). At the individual level (Figure 2C), we noted that the majority of participants had an increase in mid‐frequency alpha power during the awake resting state, but there were no significant consistent changes during the closed‐eye state. Furthermore, there was no significant difference in the power of medium alpha during the eyes‐open resting state before training when comparing the HIIT group with its corresponding control group (Figure 2D). This difference in mid‐frequency alpha power appears more pronounced when visualized in the power spectrum (Figure 2E).

### 3.3 Significant Increase in Medium Alpha Power in Closed‐Eye State in the MICT Group

The yoga group underwent an 8‐week daily fixed MICT training (Figure 3A). We found that in the closed‐eye state, there was a significant increase in low‐frequency alpha band power (~8.4 Hz, low alpha) in bilateral temporal regions (ROI‐2) and a significant decrease in high‐frequency alpha band (~11.9 Hz, high alpha) power (ROI‐3). These changes were observed exclusively during the closed‐eye state and were not significant differences in other frequency bands or in the open‐eye state (Figure 3B). At the individual level (Figure 3C), we found that most participants had increased low‐frequency alpha power and decreased high‐frequency alpha power in the open‐eye resting state, while there was no significant consistent change in the open‐eye state. Before training, there was a weak significant difference in the low‐frequency alpha power in the closed‐eye resting state between the MICT group and its corresponding control group, and there was no significant difference in the high‐frequency alpha power (Figure 3D). The difference between low‐ and high‐frequency alpha power becomes more apparent when visualized in the spectrum (Figure 3E).

### 3.4 Physical Changes and Their Relationship With Alpha‐Band Activities Change

**FIGURE 4:**
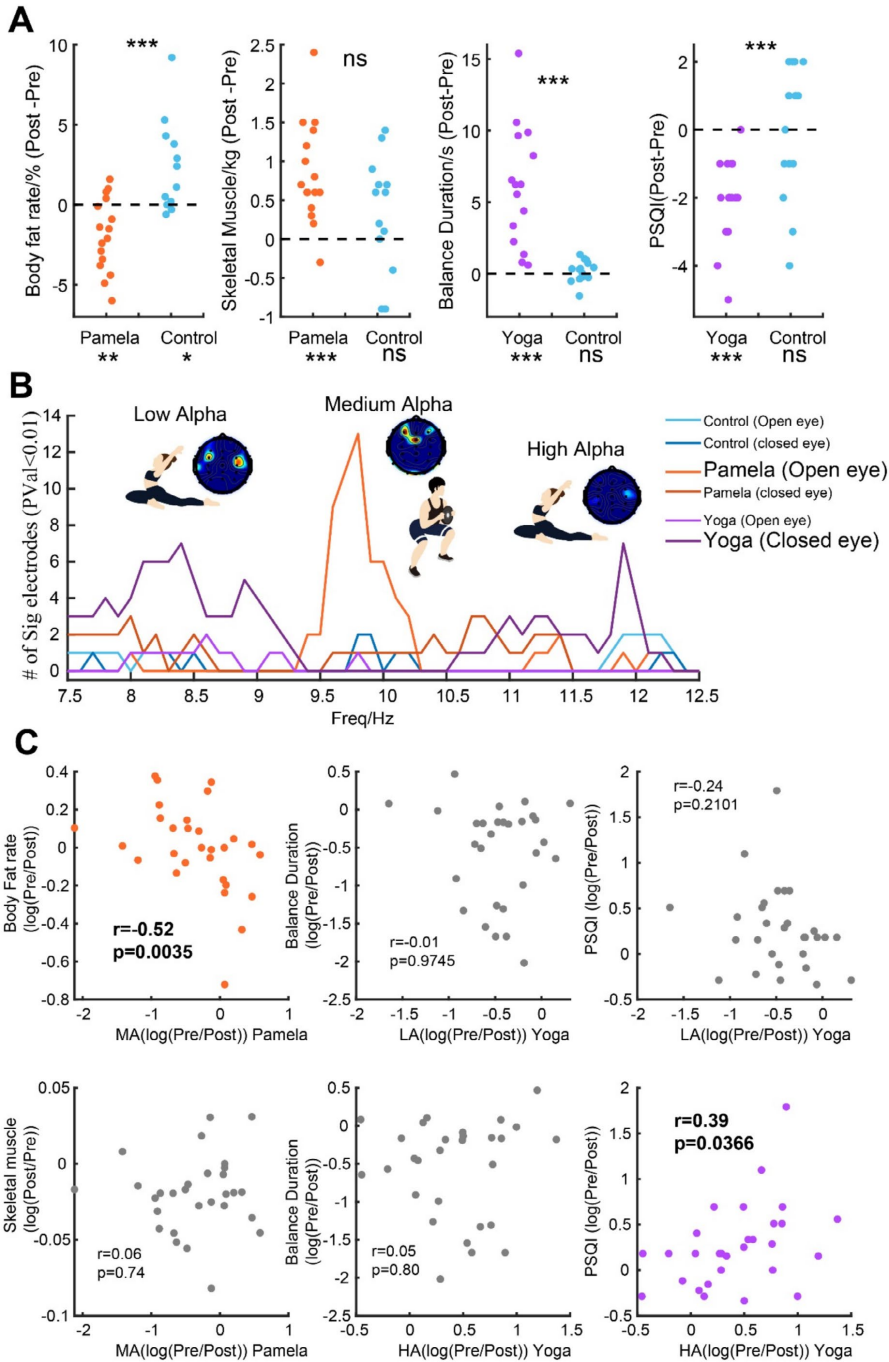
Change of physiological indicators for 8‐week HIIT and MICT training and a summary of the change of alpha‐band activities before and after 8‐week HIIT and MICT training. (A) Change of physiological and behavioral indicators (body fat percentage, skeletal muscle mass, body balance duration, and Pittsburgh Sleep Quality Index (PSQI)) in different groups. The left two panels are the comparison of that in the HIIT and corresponding control group. The right two panels are the comparison of that in the yoga and corresponding control group. Beyond the comparison of two groups, the paired *t*‐test was also used to test the difference in week 0 and week 8 in each group. The significance is shown below with names. (B) Number of significant electrodes (*p* < 0.01) in power in the alpha band compared to week 0 and 8 in open and closed eye states in HIIT, yoga, and each corresponding control group. (C) Scatter plot of change of physiological indicators and change of multiple alpha band activities (LA, MA, and HA).

In the HIIT group, compared to its corresponding control group, there was a significant decrease in body fat percentage (*p* < 0.001; Figure 4A first panel on the left), and a notable increase in skeletal muscle mass (*p* < 0.001; Figure 4A second panel on the left). Conversely, in the MICT group, there was a significant increase in body balance duration (*p* < 0.001; Figure 4A second panel on the right), and sleep quality (*p* < 0.001; Figure 4A first panel on the right) compared to its corresponding control group. We then explored whether these indicators have any relationship with the multiple alpha‐band activities (Figure 4B). We found that different physiological or behavioral measures exhibit various relationships with activities in different alpha frequency bands (Figure 4C). Specifically, changes in medium alpha activity showed a significant negative correlation (*r* = −0.52, *p* = 0.0035) with changes in body fat percentage (higher medium alpha activity corresponds to lower body fat percentage). On the other hand, changes in high alpha activity exhibited a significant positive correlation (*r* = 0.39, *p* = 0.0366) with changes in sleep quality (higher high alpha activity corresponds to better sleep quality).

## 4 Discussion

Our research, for the first time, distinguishes the effects of long‐term moderate‐ and high‐intensity exercise on neural oscillation activities in the brain under different resting states, especially proposing that different sub‐frequencies within the alpha frequency band may be indicative of distinct exercise functions, which is summarized in Table 1. For individuals who underwent long‐term HIIT, we observed several changes. First, their body fat percentage significantly decreased (Figure 4 first panel on the left), and their muscle mass significantly increased (Figure 4A second panel on the left). These physical changes were paralleled by alterations in brain activity, mainly manifested in the alpha frequency band activity within the mid‐frequency of the frontal–parietal region during the open‐eye resting state (Figure 2B, Figure 4D). For individuals who underwent long‐term MICT training, the duration of maintained balance on one leg significantly increased (Figure 4A second panel on the right), and the sleep quality improved (Figure 4A first panel on the right). Correspondingly, changes in neural response mainly occurred in the alpha frequency band activity in the low and high frequencies of the bilateral frontal‐temporal regions during the closed‐eye resting state (Figure 3B, Figure 4B). The correlation analysis (Figure 4C) further illustrates that the brain activity during the closed‐eye resting state suggests the potential benefits of long‐term MICT training for improving sleep quality, while the changes during the open‐eye resting state suggest the potential advantage of HIIT in reducing body fat rate.

**TABLE 1:** Demographic and physiological characteristics.

Items	Pamela	Control‐P	X^2^/*t*	*p*	Yoga	Control‐Y	X^2^/*t*	*p*
Age	19.19 ± 1.97	20.23 ± 2.12	−1.37	0.18	±	±	−0.40	0.69
Gender	14 M 2F	12 M 1F	0.18	0.67	0 M 15F	0 M 14F	na	na
Body fat rate (post‐pre)	**−1.88 ± 2.27** **	2.22 ± 2.86*	−4.30	< 0.001	—	—	—	—
Skeletal muscle (post‐pre)	**0.84 ± 0.64** ***	0.33 ± 0.74	2.00	0.056	—	—	—	—
Balance duration (post‐pre)	—	—	—	—	**6.07 ± 4.18** ***	0.20 ± 0.75	5.17	< 0.001
PSQI (post‐pre)	—	—	—	—	**−2.07 ± 1.28** ***	−0.07 ± 1.98	−3.25	0.0031
Low Alpha—Closed (post‐pre)‐Y	0.22 ± 0.39*	0.20 ± 0.28	0.18	0.86	**0.33 ± 0.24** ***	0.10 ± 0.43	1.85	0.08
Low Alpha—Open (post‐pre)‐Y	0.03 ± 0.56	0.05 ± 0.42	−0.41	0.92	0.20 ± 0.31*	0.06 ± 0.41	1.04	0.31
High alpha—Closed (post‐pre)‐Y	−0.06 ± 0.65	0.01 ± 0.39	−0.34	0.73	**−0.57 ± 0.57** **	−0.04 ± 0.56	−2.55	0.017
High alpha—Open (post‐pre)‐Y	−0.33 ± 0.78	−0.3 ± 0.70	−0.13	0.90	−0.23 ± 0.67	−0.18 ± 0.72	−0.21	0.83
Medium alpha—Open (post‐pre)‐P	**0.78 ± 0.65** ***	0.53 ± 1.78	0.52	0.61	0.24 ± 0.97	0.03 ± 0.56	0.71	0.48
Medium alpha—Closed (post‐pre)‐P	−0.12 ± 0.97	−0.35 ± 1.16	0.58	0.57	−0.14 ± 0.71	−0.27 ± 1.06	0.40	0.69

*Note:* Paired *t*‐test for comparing pre and post. After FDR correction, the significance of paired *t*‐test is shown in bold. Pre means week 0 before exercise, and post means week 8 after exercise.

* *p* < 0.05.

** *p* < 0.01.

*** *p* < 0.001.

### 4.1 Oscillatory Mechanism of Long‐Term High Intensity Exercise (HIIT)

Previous research has hinted at a correlation between high‐intensity exercise and alpha oscillations [26], but the specific frequency bands involved remained unclear. Our results indicate that after 8 weeks of HIIT training, participants showed a significant increase in the medium alpha frequency band during the awake state, with the main brain region affected being the frontoparietal area. This suggests that 8 weeks of high‐intensity Pamela exercise primarily enhances executive functions related to real‐life work or study, which are typically more active during the eyes‐open state. Prior studies have shown that greater alpha power in the frontal–parietal region is associated with higher target detection [68], which is highly relevant to selective attention. Therefore, people engaging in long‐term high‐intensity exercise may benefit from better daytime work efficiency. Nevertheless, we acknowledge that we currently lack direct evidence to support these explanations; further research directly measuring these resulting cognitive enhancements and their duration is necessary.

It is also notable that we found a relationship between medium alpha and body fat percentage. To the best of our knowledge, we have not come across any previous literature reporting an association between medium alpha activity and body fat percentage. However, existing studies indicate that activation in the prefrontal cortex increases after exercise [69], and body fat percentage has been found to be predictive of exercise‐related prefrontal activation and behavioral performance. Interestingly, our study also observed significant changes in the prefrontal region, and we further suggest that this may be related to medium alpha activity.

### 4.2 Oscillatory Mechanism of Long‐Term Medium Intensity Exercise (MICT)

Previous studies have generally posited that yoga, a type of MICT training, intensifies alpha oscillations [70, 71, 72]. However, our study found that the strength of alpha oscillations does not always significantly increase in all cases (e.g., when the eyes are open). When comparing the frequency of alpha sub‐bands in a closed‐eye state, only low alpha is consistent with previous findings [73], suggesting that the increase in low alpha represents an increase in the participants' relaxation levels. Prior research also consistently showed that meditation can have a significant impact on EEG alpha oscillations [74, 75, 76, 77]. Specifically, studies have found that various forms of meditation, such as mindfulness and focused attention (FA) meditation, are associated with increased alpha‐band activity. Future research should employ longitudinal designs and multimodal approaches to clarify alpha's role as a biomarker of meditative states and traits.

While in the high alpha frequency band, the opposite result is obtained, that is, the power decreases after 8 weeks of yoga training, which may indicate that MICT training may make it easier for people to fall asleep (Figure 4A first panel on the right). Also, the PSQI value also significantly decreased after training for the Yoga group (*p* < 0.001), but not for controls. This notion also aligns with previous sleep research, which has identified significant reductions in high alpha power during the transition from wakefulness to sleep [78], conveys the idea of which that facilitates or triggers sleep. Furthermore, this decrease in high alpha power is significantly related to long‐term memory performance [78]. This also suggests that through 8 weeks of MICT training, people's long‐term memory may improve. In addition, the significant changes in high and low alpha occurred mainly in the bilateral temporal–frontal regions, indicating that although there may have enhancement effects within the same region, their effects are not the same and may aid in conserving neural energy (i.e., a neural mechanism underpinning these fluctuations).

In sum, our findings underscore the multifaceted effects of exercise on both physical and neural outcomes. Furthermore, our findings highlight the potential benefits of tailored exercise interventions in addressing specific health and cognitive needs, offering valuable insights into optimizing exercise regimens for individuals seeking specific outcomes.

### 4.3 Limitations and Future Work

In the spatial level, our current research utilized 32‐channel EEG data collection, and we plan to use higher‐density EEG setups in the future, such as 128‐channel or 256‐channel EEG, to achieve more precise spatial resolution to further refine the functional differences between the frontal and temporal regions of the brain. Future research will use higher‐density EEG equipment to record brain signals after daily exercise to explore the association between neural oscillations and various exercise intensities in greater depth. Another limitation is that due to the participants' personal preferences and habits, the gender ratio of recruited participants in the HIIT and MICT groups was opposite. Although we did not observe a significant impact of gender on neural responses in Figure 1D, it is still important to balance the gender ratio in future experiments. Besides, due to the significant artifacts in EEG recordings during movement, this study did not record EEG during participant's physical activity. Instead, EEG recordings were conducted before and after 8 weeks of exercise in a resting state. Future research can further investigate how neural responses and brain functions change during the exercise intervention process. Meanwhile, 8weeks is not a fixed time; how different durations of intervention affect neural response is also worth investigating in the future. In the end, Yoga typically involves holding postures for extended periods, whereas Pamela consists of fast‐paced movements, which likely engage different cognitive processes, such as cognitive engagement, attentional demands, and motor control. However, in the current study, we are not yet able to distinguish such fine details, such as how specific movement characteristics in Yoga are precisely associated with different alpha‐band activities. Nevertheless, we believe these aspects are highly meaningful, and we will consider further controlling these detailed variables in future experimental designs as potential factors influencing alpha oscillations.

## Author Contributions

K.S. and C.H. conceived and designed the study. K.S., H.L., L.C., and C.H. contributed to the literature search, H.L. and L.C. contributed to data collection. C.H., H.L., and L.C. contributed to the data analysis and the interpretation of results. All authors contributed to writing the paper.

## Ethics Statement

This study was approved by the Beijing Sport University Ethics Committee (Sports Science Experiment) (Approval number: 2020073A).

## Consent

All participants in the experiments gave informed consent to participate in this study.

## Conflicts of Interest

The authors declare no conflicts of interest.

## Data Availability

The dataset and codes are available upon reasonable request to the corresponding author.

## References

[1] A. Flöel , R. Ruscheweyh , K. Krüger , et al., “Physical Activity and Memory Functions: Are Neurotrophins and Cerebral Gray Matter Volume the Missing Link?,” NeuroImage 49 (2010): 2756–2763, 10.1016/J.NEUROIMAGE.2009.10.043. 19853041

[2] S. Wang and S. E. Gathercole , “Working Memory Deficits in Children With Reading Difficulties: Memory Span and Dual Task Coordination,” Journal of Experimental Child Psychology 115 (2013): 188–197, 10.1016/J.JECP.2012.11.015. 23403228

[3] K. I. Erickson , M. W. Voss , R. S. Prakash , et al., “Exercise Training Increases Size of Hippocampus and Improves Memory,” Proceedings of the National Academy of Sciences of the United States of America 108 (2011): 3017–3022, 10.1073/PNAS.1015950108. 21282661 PMC3041121

[4] F. Yu , N. W. Nelson , K. Savik , J. F. Wyman , M. Dysken , and U. G. Bronas , “Affecting Cognition and Quality of Life via Aerobic Exercise in Alzheimer's Disease,” Western Journal of Nursing Research 35 (2013): 24–38, 10.1177/0193945911420174. 21911546 PMC5696626

[5] A. V. Tyndall , M. H. Davenport , B. J. Wilson , et al., “The Brain‐In‐Motion Study: Effect of a 6‐Month Aerobic Exercise Intervention on Cerebrovascular Regulation and Cognitive Function in Older Adults,” BMC Geriatrics 13 (2013): 21, 10.1186/1471-2318-13-21. 23448504 PMC3598522

[6] J. P. Folland and A. G. Williams , “The Adaptations to Strength Training: Morphological and Neurological Contributions to Increased Strength,” Sports Medicine 37 (2007): 145–168, 10.2165/00007256-200737020-00004. 17241104

[7] C. Fiuza‐Luces , A. Santos‐Lozano , M. Joyner , et al., “Exercise Benefits in Cardiovascular Disease: Beyond Attenuation of Traditional Risk Factors,” Nature Reviews. Cardiology 15 (2018): 731–743, 10.1038/S41569-018-0065-1. 30115967

[8] S. F. M. Chastin , M. De Craemer , K. De Cocker , et al., “How Does Light‐Intensity Physical Activity Associate With Adult Cardiometabolic Health and Mortality? Systematic Review With Meta‐Analysis of Experimental and Observational Studies,” British Journal of Sports Medicine 53 (2019): 370–376, 10.1136/BJSPORTS-2017-097563. 29695511 PMC6579499

[9] S. Jiang , C. Han , Y. Ma , J. Ji , G. Chen , and Y. Guo , “Temporal Dynamic Effects of Meteorological Factors and Air Quality on the Physical Health of the Older Adults in Shenzhen, China,” Frontiers in Public Health 12 (2024): 1289253, 10.3389/fpubh.2024.1289253. 38510362 PMC10951054

[10] R. Gloeckl , T. Schneeberger , I. Jarosch , and K. Kenn , “Pulmonary Rehabilitation and Exercise Training in Chronic Obstructive Pulmonary Disease,” Deutsches Ärzteblatt International 115 (2018): 117–123, 10.3238/ARZTEBL.2018.0117. 29526182 PMC5852307

[11] A. Büssing , A. Michalsen , S. B. S. Khalsa , S. Telles , and K. J. Sherman , “Effects of Yoga on Mental and Physical Health: A Short Summary of Reviews,” Evidence‐Based Complementary and Alternative Medicine 2012 (2012): 165410, 10.1155/2012/165410. 23008738 PMC3447533

[12] H. Cramer , R. Lauche , J. Langhorst , and G. Dobos , “Yoga for Rheumatic Diseases: A Systematic Review,” Rheumatology (Oxford, England) 52 (2013): 2025–2030, 10.1093/RHEUMATOLOGY/KET264. 23934220

[13] V. Gremeaux , J. Drigny , A. Nigam , et al., “Long‐Term Lifestyle Intervention With Optimized High‐Intensity Interval Training Improves Body Composition, Cardiometabolic Risk, and Exercise Parameters in Patients With Abdominal Obesity,” American Journal of Physical Medicine & Rehabilitation 91 (2012): 941–950, 10.1097/PHM.0B013E3182643CE0. 22854902

[14] D. Wen , T. Utesch , J. Wu , et al., “Effects of Different Protocols of High Intensity Interval Training for VO2max Improvements in Adults: A Meta‐Analysis of Randomised Controlled Trials,” Journal of Science and Medicine in Sport 22 (2019): 941–947, 10.1016/J.JSAMS.2019.01.013. 30733142

[15] T. A. Calverley , S. Ogoh , C. J. Marley , et al., “HIITing the Brain With Exercise: Mechanisms, Consequences and Practical Recommendations,” Journal of Physiology 598 (2020): 2513–2530, 10.1113/JP275021. 32347544

[16] R. Ramírez‐Vélez , P. A. Hernández‐Quiñones , A. Tordecilla‐Sanders , et al., “Effectiveness of HIIT Compared to Moderate Continuous Training in Improving Vascular Parameters in Inactive Adults,” Lipids in Health and Disease 18, no. 1 (2019): 42, 10.1186/S12944-019-0981-Z. 30717757 PMC6362599

[17] J. L. Taylor , D. J. Holland , S. E. Keating , et al., “Short‐Term and Long‐Term Feasibility, Safety, and Efficacy of High‐Intensity Interval Training in Cardiac Rehabilitation: The FITR Heart Study Randomized Clinical Trial,” JAMA Cardiology 5 (2020): 1382–1389, 10.1001/JAMACARDIO.2020.3511. 32876655 PMC7489382

[18] S. Makeig , M. Westerfield , T. P. Jung , et al., “Dynamic Brain Sources of Visual Evoked Responses,” Science 295, no. 5555 (2002): 690–694, 10.1126/science.1066168. 11809976

[19] B. Wang , M. Li , N. Haihambo , et al., “Characterizing Major Depressive Disorder (MDD) Using Alpha‐Band Activity in Resting‐State Electroencephalogram (EEG) Combined With MATRICS Consensus Cognitive Battery (MCCB),” Journal of Affective Disorders 355 (2024): 254–264, 10.1016/j.jad.2024.03.145. 38561155

[20] T. Zikereya , Y. Lin , Z. Zhang , I. Taguas , K. Shi , and C. Han , “Different Oscillatory Mechanisms of Dementia‐Related Diseases With Cognitive Impairment in Closed‐Eye State,” NeuroImage 304 (2024): 120945, 10.1016/J.NEUROIMAGE.2024.120945. 39586346

[21] Y. Lin , S. Huang , J. Mao , et al., “The Neural Oscillatory Mechanism Underlying Human Brain Fingerprint Recognition Using a Portable EEG Acquisition Device,” NeuroImage 294 (2024): 120637, 10.1016/J.NEUROIMAGE.2024.120637. 38714216

[22] P. Liu , C. Han , T. Zhang , et al., “Alterations of Oscillatory Activity and Cognitive Function After Aneurysmal Subarachnoid Hemorrhage,” International Journal of Surgery 111, no. 2 (2024): 1919–1928, 10.1097/JS9.0000000000002190. 39715156

[23] Y. Zhang , Z. Zhang , F. Du , et al., “Shared Oscillatory Mechanisms of Alpha‐Band Activity in Prefrontal Regions in Eyes Open and Closed State Using a Portable EEG Acquisition Device,” Scientific Reports 14 (2024): 26719, 10.1038/S41598-024-78173-0. 39496816 PMC11535223

[24] C. Han , Z. Zhang , Y. Lin , et al., “Monitoring Sleep Quality Through Low α‐Band Activity in the Prefrontal Cortex Using a Portable Electroencephalogram Device: Longitudinal Study,” Journal of Medical Internet Research 27 (2025): e67188, 10.2196/67188. 40063935 PMC11933759

[25] K. I. Erickson , C. Hillman , C. M. Stillman , et al., “Physical Activity, Cognition, and Brain Outcomes: A Review of the 2018 Physical Activity Guidelines,” Medicine and Science in Sports and Exercise 51 (2019): 1242–1251, 10.1249/MSS.0000000000001936. 31095081 PMC6527141

[26] J. Wang , X. Zhao , Y. Bi , et al., “Executive Function Elevated by Long Term High‐Intensity Physical Activity and the Regulation Role of Beta‐Band Activity in Human Frontal Region,” Cognition, Brain, & Behavior 17 (2022): 1–10, 10.1007/S11571-022-09905-Z/FIGURES/6. PMC10640436 37974584

[27] C. Han , M. Guo , X. Ke , et al., “Oscillatory Biomarkers of Autism: Evidence From the Innate Visual Fear Evoking Paradigm,” Cognition, Brain, & Behavior 17, no. 2 (2023): 459–466, 10.1007/s11571-022-09839-6. PMC10050250 37007195

[28] C. Han , X. Zhao , M. Li , et al., “Enhancement of the Neural Response During 40 Hz Auditory Entrainment in Closed‐Eye State in Human Prefrontal Region,” Cognition, Brain, & Behavior 17, no. 2 (2023): 399–410, 10.1007/s11571-022-09834-x. PMC10050539 37007205

[29] Y. Cao , C. Han , X. Peng , et al., “Correlation Between Resting Theta Power and Cognitive Performance in Patients With Schizophrenia,” Frontiers in Human Neuroscience 16 (2022): 853994, 10.3389/fnhum.2022.853994. 35529780 PMC9074816

[30] C. Han , T. Wang , Y. Wu , et al., “Compensatory Mechanism of Attention‐Deficit / Hyperactivity Disorder Recovery in Resting State Alpha Rhythms,” Frontiers in Computational Neuroscience 16 (2022): 883065, 10.3389/fncom.2022.883065. 36157841 PMC9490822

[31] D. Rudrauf , A. Douiri , C. Kovach , et al., “Frequency Flows and the Time‐Frequency Dynamics of Multivariate Phase Synchronization in Brain Signals,” NeuroImage 31 (2006): 209–227, 10.1016/j.neuroimage.2005.11.021. 16413209

[32] C. Han , R. Shapley , and D. Xing , “Gamma Rhythms in the Visual Cortex: Functions and Mechanisms,” Cognition, Brain, & Behavior 16, no. 4 (2022): 745–756, 10.1007/s11571-021-09767-x. PMC9279528 35847544

[33] C. Han , T. Wang , Y. Wu , et al., “The Generation and Modulation of Distinct Gamma Oscillations With Local, Horizontal, and Feedback Connections in the Primary Visual Cortex: A Model Study on Large‐Scale Networks,” Neural Plasticity 2021 (2021): 8874516, 10.1155/2021/8874516. 33531893 PMC7834828

[34] C. Han , T. Wang , Y. Yang , et al., “Multiple Gamma Rhythms Carry Distinct Spatial Frequency Information in Primary Visual Cortex,” PLoS Biology 19, no. 12 (2021): e3001466, 10.1371/JOURNAL.PBIO.3001466. 34932558 PMC8691622

[35] C. Han , “The Oscillating Mystery: The Effects of Forty‐Hertz Entrainment in Treating Alzheimer's Disease,” Brain‐X 1 (2023): e14, 10.1002/BRX2.14.

[36] C. Han , “Editorial: Mechanism of Neural Oscillations and Their Relationship With Multiple Cognitive Functions and Mental Disorders,” Frontiers in Neuroscience 18 (2024): 1543731, 10.3389/FNINS.2024.1543731. 39840018 PMC11747451

[37] W. Klimesch , “Alpha‐Band Oscillations, Attention, and Controlled Access to Stored Information,” Trends in Cognitive Sciences 16 (2012): 606–617, 10.1016/j.tics.2012.10.007. 23141428 PMC3507158

[38] T. A. Rihs , C. M. Michel , and G. Thut , “A Bias for Posterior Alpha‐Band Power Suppression Versus Enhancement During Shifting Versus Maintenance of Spatial Attention,” NeuroImage 44 (2009): 190–199, 10.1016/J.NEUROIMAGE.2008.08.022. 18793732

[39] A. Bollimunta , J. Mo , C. E. Schroeder , and M. Ding , “Neuronal Mechanisms and Attentional Modulation of Corticothalamic Alpha Oscillations,” Journal of Neuroscience 31 (2011): 4935–4943, 10.1523/JNEUROSCI.5580-10.2011. 21451032 PMC3505610

[40] S. Hanslmayr , J. Gross , W. Klimesch , and K. L. Shapiro , “The Role of Alpha Oscillations in Temporal Attention,” Brain Research Reviews 67 (2011): 331–343, 10.1016/j.brainresrev.2011.04.002. 21592583

[41] Y. Bagherzadeh , D. Baldauf , D. Pantazis , and R. Desimone , “Alpha Synchrony and the Neurofeedback Control of Spatial Attention,” Neuron 105 (2020): 577–587.e5, 10.1016/j.neuron.2019.11.001. 31812515

[42] R. Gulbinaite , T. Van Viegen , M. Wieling , M. X. Cohen , and R. Vanrullen , “Individual Alpha Peak Frequency Predicts 10 Hz Flicker Effects on Selective Attention,” Journal of Neuroscience 37 (2017): 10173–10184, 10.1523/JNEUROSCI.1163-17.2017. 28931569 PMC6596538

[43] F. Roux and P. J. Uhlhaas , “Working Memory and Neural Oscillations: Alpha‐Gamma Versus Theta‐Gamma Codes for Distinct WM Information?,” Trends in Cognitive Sciences 18 (2014): 16–25, 10.1016/j.tics.2013.10.010. 24268290

[44] J. J. Foster , E. M. Bsales , R. J. Jaffe , and E. Awh , “Alpha‐Band Activity Reveals Spontaneous Representations of Spatial Position in Visual Working Memory,” Current Biology 27 (2017): 3216–3223.e6, 10.1016/j.cub.2017.09.031. 29033335 PMC5661984

[45] W. Klimesch , “EEG‐Alpha Rhythms and Memory Processes,” International Journal of Psychophysiology 26 (1997): 319–340, 10.1016/S0167-8760(97)00773-3. 9203012

[46] L. Michels , M. Moazami‐Goudarzi , D. Jeanmonod , and J. Sarnthein , “EEG Alpha Distinguishes Between Cuneal and Precuneal Activation in Working Memory,” NeuroImage 40 (2008): 1296–1310, 10.1016/j.neuroimage.2007.12.048. 18272404

[47] C. Escolano , M. Aguilar , and J. Minguez , “EEG‐Based Upper Alpha Neurofeedback Training Improves Working Memory Performance,” Proceedings of the Annual International Conference of the IEEE Engineering in Medicine and Biology Society, EMBS 2011 (2011): 2327–2330, 10.1109/IEMBS.2011.6090651. 22254807

[48] R. Freunberger , R. Fellinger , P. Sauseng , W. Gruber , and W. Klimesch , “Dissociation Between Phase‐Locked and Nonphase‐Locked Alpha Oscillations in a Working Memory Task,” Human Brain Mapping 30 (2009): 3417–3425, 10.1002/hbm.20766. 19384888 PMC6870638

[49] T. Toosi , E. K. Tousi , and H. Esteky , “Learning Temporal Context Shapes Prestimulus Alpha Oscillations and Improves Visual Discrimination Performance,” Journal of Neurophysiology 118, no. 2 (2017): 771–777, 10.1152/jn.00969.2016. 28515289 PMC5539440

[50] J. Mo , Y. Liu , H. Huang , and M. Ding , “Coupling Between Visual Alpha Oscillations and Default Mode Activity,” NeuroImage 68 (2013): 112–118, 10.1016/J.NEUROIMAGE.2012.11.058. 23228510 PMC3557590

[51] T. Komiyama , R. Goya , C. Aoyama , Y. Yokota , Y. Naruse , and S. Shimegi , “The Combination of Acute Exercise and Eye Closure has a Synergistic Effect on Alpha Activity,” Scientific Reports 11, no. 1 (2021): 20186, 10.1038/S41598-021-99783-Y. 34642438 PMC8511023

[52] J. Lagopoulos , J. Xu , I. Rasmussen , et al., “Increased Theta and Alpha EEG Activity During Nondirective Meditation,” Journal of Alternative and Complementary Medicine 15 (2009): 1187–1192, 10.1089/ACM.2009.0113. 19922249

[53] E. Barzegaran , V. Y. Vildavski , and M. G. Knyazeva , “Fine Structure of Posterior Alpha Rhythm in Human EEG: Frequency Components, Their Cortical Sources, and Temporal Behavior,” Scientific Reports 7 (2017): 1–12, 10.1038/s41598-017-08421-z. 28811538 PMC5557761

[54] J. Hertel , S. J. Miller , and C. R. Denegar , “Intratester and Intertester Reliability During the Star Excursion Balance Tests,” Journal of Sport Rehabilitation 9 (2000): 104–116, 10.1123/JSR.9.2.104.

[55] D. J. Buysse , C. F. Reynolds , T. H. Monk , S. R. Berman , and D. J. Kupfer , “The Pittsburgh Sleep Quality Index: A New Instrument for Psychiatric Practice and Research,” Psychiatry Research 28 (1989): 193–213, 10.1016/0165-1781(89)90047-4. 2748771

[56] A. Delorme and S. Makeig , “EEGLAB: An Open Source Toolbox for Analysis of Single‐Trial EEG Dynamics Including Independent Component Analysis,” Journal of Neuroscience Methods 134 (2004): 9–21, 10.1016/j.jneumeth.2003.10.009. 15102499

[57] C. Han , B. Wang , G. Yang , et al., “Neural Mechanism of Orientation Selectivity for Distinct Gamma Oscillations in Cat V1,” Journal of Vision 20 (2020): 1116, 10.1167/jov.20.11.1116.

[58] D. Ge , C. Han , C. Liu , and Z. Meng , “Neural Oscillations in the Somatosensory and Motor Cortex Distinguish Dexmedetomidine‐Induced Anesthesia and Sleep in Rats,” CNS Neuroscience & Therapeutics 31, no. 2 (2025): e70262, 10.1111/cns.70262. 39963924 PMC11833454

[59] X. Zhao , B. Wang , J. Liu , et al., “Distinguishing Major Depressive Disorder From Bipolar Disorder Using Alpha‐Band Activity in Resting‐State Electroencephalogram,” Journal of Affective Disorders 376 (2025): 333–340, 10.1016/j.jad.2025.02.032. 39961442

[60] X. Zhao , M. Li , N. Haihambo , et al., “Changes in Temporal Properties of Notifiable Infectious Disease Epidemics in China During the COVID‐19 Pandemic: Population‐Based Surveillance Study,” JMIR Public Health and Surveillance 8, no. 6 (2022): 1–12, 10.2196/35343. PMC9231598 35649394

[61] X. Zhao , M. Li , N. Haihambo , et al., “Periodic Characteristics of Hepatitis Virus Infections From 2013 to 2020 and Their Association With Meteorological Factors in Guangdong, China: Surveillance Study,” JMIR Public Health and Surveillance 9 (2023): e45199, 10.2196/45199. 37318858 PMC10337419

[62] R. M. Anderson , B. T. Grenfell , and R. M. May , “Oscillatory Fluctuations in the Incidence of Infectious Disease and the Impact of Vaccination: Time Series Analysis,” Journal of Hygiene (London) 93 (1984): 587–608, 10.1017/S0022172400065177. PMC2129464 6512259

[63] B. Cazelles , M. Chavez , G. C. De Magny , J. F. Guégan , and S. Hales , “Time‐Dependent Spectral Analysis of Epidemiological Time‐Series With Wavelets,” Journal of the Royal Society Interface 4 (2007): 625–636, 10.1098/RSIF.2007.0212. 17301013 PMC2373388

[64] C. Han , M. Li , N. Haihambo , Y. Cao , and X. Zhao , “Enlightenment on Oscillatory Properties of 23 Class B Notifiable Infectious Diseases in the Mainland of China From 2004 to 2020,” PLoS One 16 (2021): e0252803, 10.1371/journal.pone.0252803. 34106977 PMC8189525

[65] C. Han , M. Li , N. Haihambo , and P. Babuna , “Mechanisms of Recurrent Outbreak of COVID‐19: A Model‐ Based Study,” Nonlinear Dynamics 106 (2021): 1169–1185, 10.1007/s11071-021-06371-w. 33758464 PMC7972336

[66] Y. Cao , M. Li , N. Haihambo , et al., “Oscillatory Properties of Class C Notifiable Infectious Diseases in China From 2009 to 2021,” Frontiers in Public Health 10 (2022): 903025, 10.3389/fpubh.2022.903025. 36033737 PMC9402928

[67] H. Bokil , P. Andrews , J. E. Kulkarni , S. Mehta , and P. P. Mitra , “Chronux: A Platform for Analyzing Neural Signals,” Journal of Neuroscience Methods 192, no. 1 (2010): 146–151, 10.1016/j.jneumeth.2010.06.020. 20637804 PMC2934871

[68] J. Jia , F. Fang , and H. Luo , “Selective Spatial Attention Involves Two Alpha‐Band Components Associated With Distinct Spatiotemporal and Functional Characteristics,” NeuroImage 199 (2019): 228–236, 10.1016/J.NEUROIMAGE.2019.05.079. 31154048

[69] J. Crum , F. Ronca , G. Herbert , et al., “Body Fat Predictive of Acute Effects of Exercise on Prefrontal Hemodynamics and Speed,” Neuropsychologia 196 (2024): 108805, 10.1016/J.NEUROPSYCHOLOGIA.2024.108805. 38340963

[70] S. Yamamoto , Y. Kitamura , N. Yamada , Y. Nakashima , and S. Kuroda , “Medial Profrontal Cortex and Anterior Cingulate Cortex in the Generation of Alpha Activity Induced by Transcendental Meditation: A Magnetoencephalographic Study,” Acta Medica Okayama 60 (2006): 51–58, 10.18926/AMO/30752. 16508689

[71] S. Chandra , G. Sharma , A. Mittal , and D. Jha , “Effect of Sudarshan Kriya (Meditation) on Gamma, Alpha, and Theta Rhythm During Working Memory Task,” International Journal of Yoga 9 (2016): 72–76, 10.4103/0973-6131.171715. 26865775 PMC4728963

[72] R. M. Vivot , C. Pallavicini , F. Zamberlan , D. Vigo , and E. Tagliazucchi , “Meditation Increases the Entropy of Brain Oscillatory Activity,” Neuroscience 431 (2020): 40–51, 10.1016/J.NEUROSCIENCE.2020.01.033. 32032666

[73] L. I. Aftanas and S. A. Golocheikine , “Human Anterior and Frontal Midline Theta and Lower Alpha Reflect Emotionally Positive State and Internalized Attention: High‐Resolution EEG Investigation of Meditation,” Neuroscience Letters 310 (2001): 57–60, 10.1016/S0304-3940(01)02094-8. 11524157

[74] J. Gao , R. Sun , H. K. Leung , et al., “Increased Neurocardiological Interplay After Mindfulness Meditation: A Brain Oscillation‐Based Approach,” Frontiers in Human Neuroscience 17 (2023): 1008490, 10.3389/FNHUM.2023.1008490. 37405324 PMC10315629

[75] S. Katyal and P. Goldin , “Alpha and Theta Oscillations Are Inversely Related to Progressive Levels of Meditation Depth,” Neuroscience of Consciousness 2021, no. 1 (2021): niab042, 10.1093/NC/NIAB042. 34858638 PMC8633885

[76] A. T. Duda , A. R. Clarke , R. J. Barry , and F. M. De Blasio , “Mindfulness Meditation Is Associated With Global EEG Spectral Changes in Theta, Alpha, and Beta Amplitudes,” International Journal of Psychophysiology 206 (2024): 112465, 10.1016/J.IJPSYCHO.2024.112465. 39557128

[77] D. J. Lee , E. Kulubya , P. Goldin , A. Goodarzi , and F. Girgis , “Review of the Neural Oscillations Underlying Meditation,” Frontiers in Neuroscience 12 (2018): 178, 10.3389/FNINS.2018.00178. 29662434 PMC5890111

[78] W. Klimesch , “EEG Alpha and Theta Oscillations Reflect Cognitive and Memory Performance: A Review and Analysis,” Brain Research Reviews 29 (1999): 169–195, 10.1016/S0165-0173(98)00056-3. 10209231

